# Application of Prospective ECG-Gated High-Pitch 128-Slice Dual-Source CT Angiography in the Diagnosis of Congenital Extracardiac Vascular Anomalies in Infants and Children

**DOI:** 10.1371/journal.pone.0115793

**Published:** 2014-12-29

**Authors:** Pei Nie, Guangjie Yang, Ximing Wang, Yanhua Duan, Wenjian Xu, Haiou Li, Ting Cao, Xuejun Liu, Xiaopeng Ji, Zhaoping Cheng, Anbiao Wang

**Affiliations:** 1 Department of Radiology, the Affiliated Hospital of Qingdao University, Qingdao, Shandong, China; 2 Department of Nuclear Medicine, Qilu Hospital, Shandong University, Jinan, Shandong, China; 3 Shandong Provincial Key Laboratory of Diagnosis and Treatment of Cardio-Cerebral Vascular Diseases, Shandong Medical Imaging Research Institute, Jinan, Shandong, China; 4 Department of Cardiovascular Surgery, Shandong Provincial Hospital, Jinan, Shandong, China; Sapienza University of Rome, Italy

## Abstract

**Purpose:**

To investigate the value of prospective ECG-gated high-pitch 128-slice dual-source CT (DSCT) angiography in the diagnosis of congenital extracardiac vascular anomalies in infants and children in comparison with transthoracic echocardiography (TTE).

**Methods:**

Eighty consecutive infants or children clinically diagnosed of congenital heart disease and suspected with extracardiac vascular anomaly were enrolled, and 75 patients were finally included in this prospective study. All patients underwent prospective ECG-gated high-pitch DSCT angiography after TTE with an interval of 1–7 days. The diagnostic accuracy and sensitivity of high-pitch DSCT angiography and TTE were compared according to the surgical/CCA findings. The image quality of DSCT was assessed using a five-point scale. The effective radiation dose (ED) was calculated.

**Results:**

A total of 17 congenital heart diseases and 162 separate extracardiac vascular anomalies were confirmed by surgical/CCA findings in 75 patients. The diagnostic accuracy of high-pitch DSCT angiography and TTE was 99.67% and 97.89%, respectively. The sensitivity of high-pitch DSCT angiography and TTE was 97.53% and 79.62%, respectively. There was significant difference regarding to the diagnostic accuracy and the sensitivity between high-pitch DSCT angiography and TTE (χ^2^ = 23.561 and 28.013, P<0.05). The agreement on the image quality scoring of DSCT between the two observers was excellent (κ = 0.81), and the mean score of image quality was 4.1±0.7. The mean ED of DSCT was 0.29±0.08 mSv.

**Conclusions:**

Prospective ECG-gated high-pitch 128-slice DSCT angiography with low radiation dose and high diagnostic accuracy has higher sensitivity compared to TTE in the detection of congenital extracardiac vascular anomalies in infants and children.

## Introduction

Precise and comprehensive evaluation of the intracardiac and extracardiac deformities in congenital heart diseases (CHD) is critical for surgical plan. Transthoracic echocardiography (TTE) is the first-line option for children with CHD because of its availability, safety and capacity to provide hemodynamic parameters using Doppler flow studies. TTE combined with the Doppler flow imaging has the advantage in the diagnosis of intracardiac deformities, especially the small atrial septal defect and ventricular septal defect. However, TTE is not robust for evaluating extracardiac structures because it is limited by its acoustic window and relatively lower spatial resolution [1]–[4].

The advanced multi-detector CT (MSCT) technology with improved spatial and temporal resolution has made it an effective tool in the assessment of CHD in infants and children [5]–[7]. The value of MSCT in the evaluation of the thoracic vascular anomalies has been widely confirmed. As this imaging modality can not only provide cardiovascular information, but also offer additional information about the airway and lung parenchyma, resulting in a greater anatomic coverage. A recent introduced dual-source CT (DSCT) system (Definition Flash, Siemens Healthcare, Forchheim, Germany) provides the high-pitch scan technique. In this high-pitch mode, data acquisition is prospectively triggered with the ECG. With a very high pitch of 3.4 and a table speed of 460 mm/s, the entire heart can be covered within one single cardiac cycle. Due to the fast, non-overlapping spiral data acquisition, the radiation dose was consequently reduced [8], [9].

The prospective ECG-gated sequential technique has been proven one of the most effective strategies to lower the radiation dose. The sequential mode allows high diagnostic accuracy in the assessment of intracardiac, extracadiac and coronary anatomies in children with CHD, and the image quality is seldom influenced by the variability of the heart rates [10]–[13]. Several studies have found that the new high-pitch mode is feasible in adults with stable and low heart rates below 65 or 70 beats per minute (bpm) [14]–[16], and its initial experience in children has been reported. With the relatively high and irregular heart rates in children with CHD, the starting position of the data acquisition in high-pitch mode usually could not correspond to the preselected starting phase, and sometimes the R wave might be covered within the acquisition window, resulting in the poor image quality of the intracardiac and coronary structures [17]. In our previous studies on high-pitch mode, we found that the image quality of extracardiac great vessels had not been influenced by the variability of heart rate, and the high-pitch mode significantly lowered the radiation dose as compared to the sequential mode. Therefore, the high-pitch mode has advantages over sequential mode in demonstrating extracardiac vascular anomalies.

The purpose of this study was to investigate the value of prospective ECG-gated high-pitch 128-slice DSCT angiography in the diagnosis of congenital extracardiac vascular anomalies in infants and children in comparison with TTE.

## Materials and Methods

### Patients

This study received approval from the institutional review board of Shandong Medical Imaging Research Institute and written informed consent was obtained from the parents of all patients. We have obtained written consent from the parents of the patients for publication of medical images used in the Figures.

From March 2013 to January 2014, 80 consecutive patients clinically diagnosed CHD with suspected extracardiac vascular anomalies were enrolled in this study. Exclusion criteria were nephropathy (n = 3) or hypersensitivity to iodinated contrast (n = 2), and 75 patients (male 43, female 32; mean age: 16.84±19.4 months, range: 1–96 months; mean weight: 9.02±4.61 kg, range: 4–28 kg; mean heart rate: 119.73±15.10 bpm, range: 82–149 bpm) were finally included. We conducted prospective ECG-gated high-pitch DSCT angiography on 75 patients after routine TTE examinations with a time interval of 1–7 days. All the patients underwent successful scanning without complications. The mean scan time was 0.37±0.06 seconds (range: 0.23–0.52 seconds). Surgery was performed in 46 patients, and conventional cardiac angiography (CCA) was performed in 29 patients.

### DSCT protocol

All examinations were performed on a 128-slice DSCT scanner (Somatom Definition Flash, Siemens Healthcare, Forchheim, Germany). Short-term sedation was achieved with oral administration of chloral hydrate. All patients were free-breathing. The scans were performed in cranio-caudal direction from the thoracic outlet to the bottom of the heart.

Iodinated contrast medium (Schering Ultravist, Iopromide, 350 mg I/mL, Berlin, Germany) was injected via peripheral veins at a volume of 1.5 mL/kg body weight. The injection time of contrast medium was 15 seconds. Injection rate was calculated at total injected volume of contrast medium divided by 15 seconds. In order to reduce the artifact of contrast medium in superior vena cava, a saline chaser of 1.0 mL/kg body weight was injected at the same rate of contrast medium. A “manual” bolus-tracking technique was used, the monitoring section was set at the four chambers of the heart and the ROI was placed at the air outside the thorax. Monitoring started 18 seconds after injection. The acquisition was manually triggered at the moment that the contrast medium opacification within both the right heart and left heart was achieved and the contrast medium artifact in the right atrium began to disappear.

The high-pitch mode was used. Data acquisition was prospectively ECG-triggered starting at 10% of the R-R interval using a pitch of 3.4. CT parameters were as follows: 0.28 s gantry rotation time, 2×64×0.6 mm detector collimation, a slice collimation 2×128×0.6 mm by z-flying focal spot technique, 80 kV tube voltage and weight adapted setting for tube current (60 mAs/rotation for patients <5 kg body weight, 60–79 mAs/rotation for patients 5–10 kg body weight, 80–120 mAs/rotation for patients>10 kg body weight).

### DSCT data post-processing and analysis

Images were reconstructed with a slice thickness of 0.75 mm and increment of 0.5 mm using a medium smooth-tissue convolution kernel (B26f). All images were transferred to an external workstation (Multiple Modality Workplace, Siemens Healthcare, Forchheim, Germany) for post-processing. Multiplanar reformation (MPR), maximum intensity projection (MIP) and volume rendering (VR) were used for image interpretation.

Blinded to the results of TTE, surgical and/or CCA findings, two cardiac radiologists with more than 5 years' experience interpreted the image quality using a 5-grade scoring system (5, excellent; 4, good; 3, fair; 2, insufficient for complete evaluation; 1, not interpretable) [18]. Consensus agreement was achieved between the two observers if disagreement existed. Grades 3, 4 and 5 were considered sufficient for complete diagnosis.

### Radiation dose estimations

The volume CT dose index (CTDIvol) and dose-length product (DLP) were obtained from the CT system and the effective radiation dose (mSv) was calculated from the DLP (mGy·cm) multiplied by 2.3 to adapt it to the 16-cm phantom (the DLP for the body surface area was given for a 32-cm phantom on the scanner protocol and the conversion factor of 2.3 is scanner specific for paediatric examinations at 80 kV as provided by the manufacturer). The corrected DLP value was then multiplied by the specific conversion coefficients given for a 16-cm phantom: 0.039 mSv/[mGy·cm] for children up to 4 months, 0.026 mSv/[mGy·cm] between 4 months and 1 year of age, and 0.018 mSv/[mGy·cm] between 1 year and 6 years of age [12], [13].

### Transthoracic echocardiography

All patients underwent two-dimensional TTE and Doppler Flow from the parasternal, apical, subxiphoid and suprasternal approaches using a SONOS 7500 ultrasound system (Philips Medical System, Bothell, WA). The examinations were performed by an experienced echocardiographic technician, and the data were evaluated by a trained pediatrician.

### Statistics

Statistical analysis was performed by SPSS 17.0 software (SPSS, Chicago, IL, USA). Results were expressed as means ± standard deviations for quantitative variables and as frequencies or percentages for categorical variables. With surgical and/or CCA results as the standard, the diagnostic accuracy and sensitivity of DSCT and TTE for the separate cardiovascular abnormalities was respectively calculated. Comparative analysis of the diagnostic accuracy and sensitivity between DSCT angiography and TTE was obtained using the non-parametric chi–square test. Interobserver agreement on grades of image quality was assessed by kappa statistics (*κ*>0.81, excellent agreement; *κ* = 0.61–0.80, good agreement). P<0.05 was considered statistically significant.

## Results

A total of 17 CHDs and 162 separate extracardiac vascular anomalies were confirmed by surgical/CCA findings in 75 patients. Twenty-three abnormalities at the cardiac-vascular junction included 2 transposition of the great arteries, 2 double outlet right ventricles, 9 overriding aortas, 3 truncus arteriosus (type I), 4 partial anomalous pulmonary venous returns (PAPVR) and 3 total anomalous pulmonary venous returns (TAPVR, including 1 supracardiac type, 1 cardiac type and 1 mixed type). One hundred and thirty-nine extracardiac anomalies included 2 aortopulmonary windows, 2 bicuspid aortic valves (BAV), 5 cases of aortic dysplasia, 2 supravalvular aortic stenoses, 6 right aortic arches, 4 double aortic arches, 23 coarctations of aorta (CoA), 4 interruptions of the aortic arch (IAA, including 2 A type and 2 B type), 5 cases of pulmonary artery atresia (4 I type and 1 II type), 10 pulmonary artery stenoses, 20 dilated pulmonary arteries, 2 cases of hemitruncus arteriosus, 2 cases of absence of a pulmonary artery, 7 pulmonary slings, 22 cases of patent ductus arteriosus (PDA), 12 major aortopulmonary collateral arteries (MAPCAs), 8 cases of persistent left superior vena cava (PLSVC) and 3 coronary artery anomalies (1 single coronary artery, 1 coronary-cameral fistula and 1 abnormal origin of the coronary artery).

### Diagnostic performance

**Figure 1:**
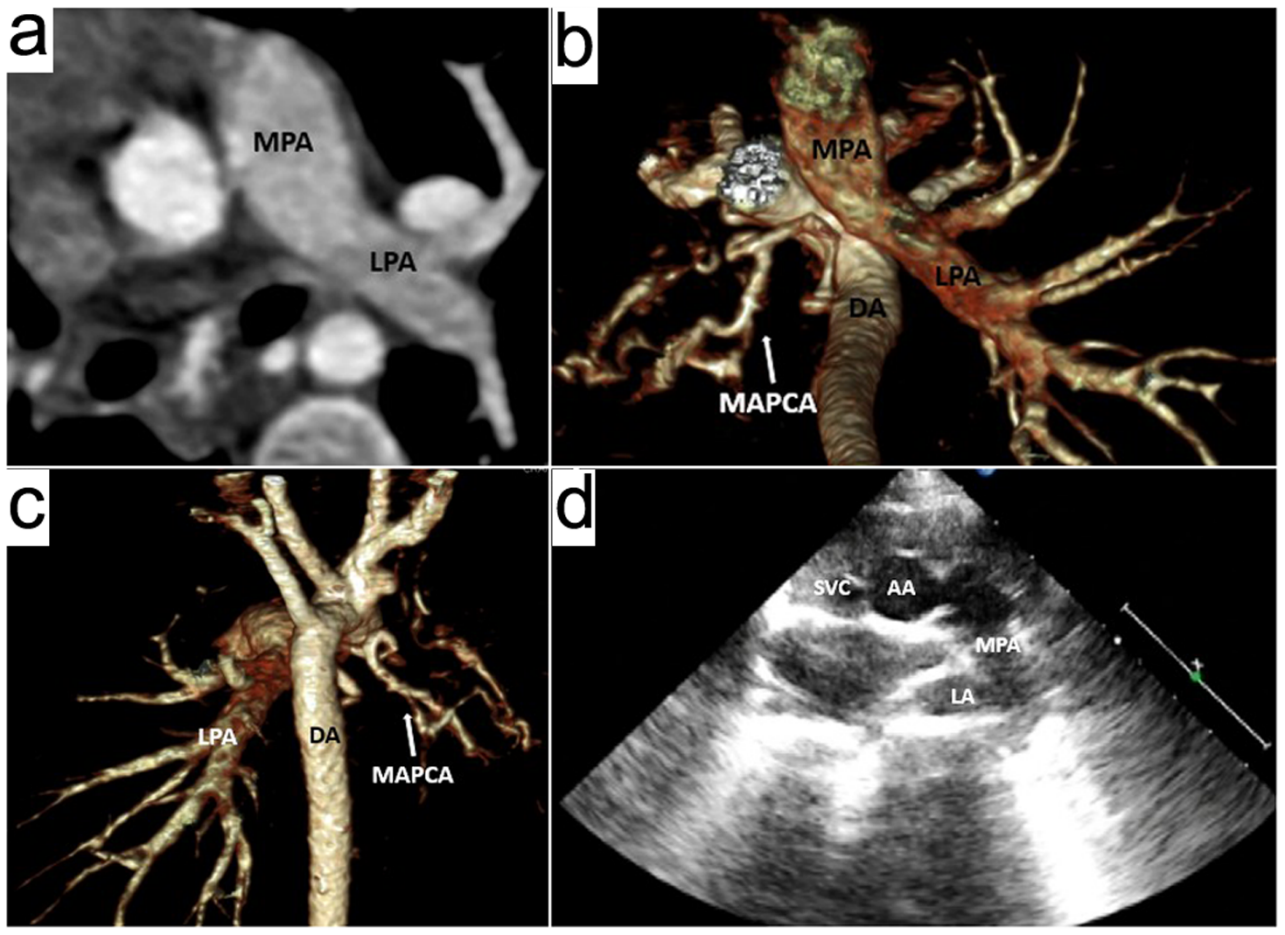
An 18-months boy with absence of the right pulmonary artery and major aortopulmonary collateral artery (MAPCA). Prospective ECG-triggering high-pitch DSCT angiography was performed at 80 kV and 80 mAs/rotation (effective radiation dose, 0.29 mSv). (a) Axial multiplanar reformatted image shows the main pulmonary artery (MPA) continues to the left pulmonary artery (LPA). (b) Volume-rendered (VR) image (inferior view) and (c) VR image (posterior view) show the absence of the right pulmonary artery (RPA), and the right lung is supplied by a MAPCA arising from the descending aorta (DA). (d) Two dimensional echocardiography from the parasternal approach mistakes the top of left atrium (LA) for RPA, and the LPA is not shown as being obscured by the aerated lung. This case was misdiagnosed as pulmonary sling on TTE. AA =  ascending aorta, SVC =  superior vena cava.

**Figure 2:**
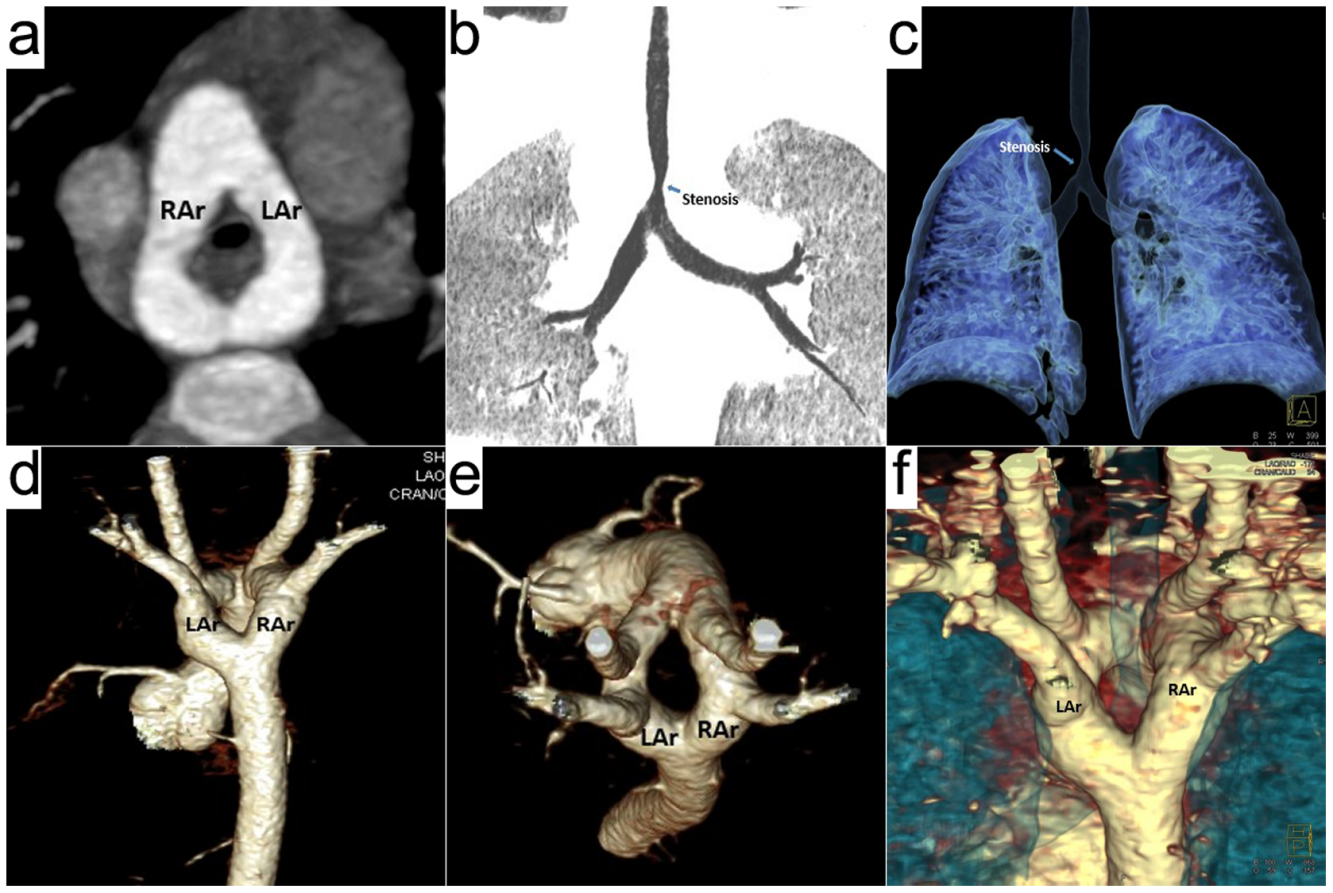
A 20-months boy with double aortic arch and tracheal stenosis. Prospective ECG-triggering high-pitch DSCT angiography was performed at 80 kV and 80 mAs/rotation (effective radiation dose, 0.21 mSv). (a) Thin-section axial MIP, (d) volume-rendered (VR) image (posterior view) and (e) VR image (superior view) show the vascular ring formed by two aortic arches. (b) Thin-section coronal minIP, (c) airway VR image and (f) airway and vascular VR image show the tracheal stenosis compressed by the vascular ring. LAr  =  left aortic arch, RAr  =  right aortic arch.

**Figure 3:**
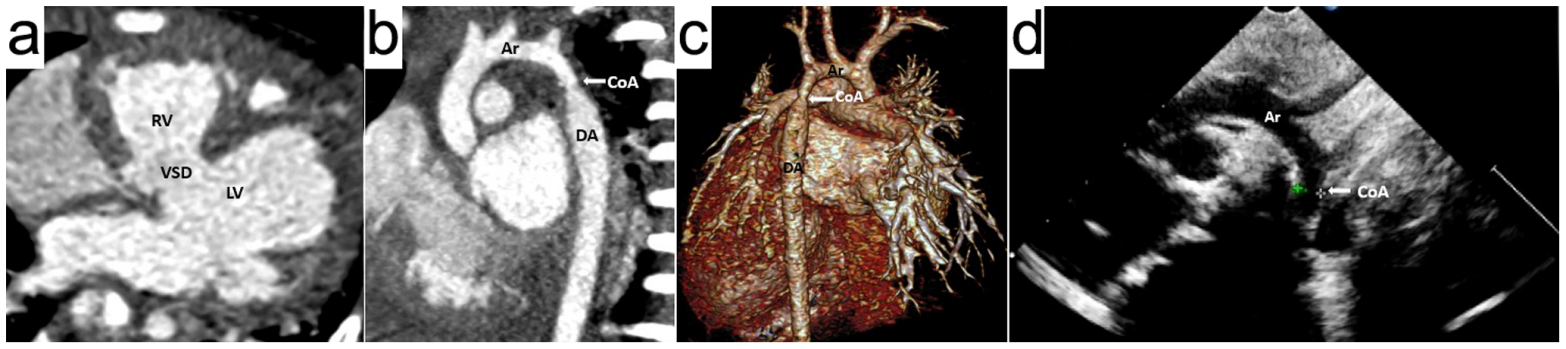
A 6-months girl with coarctation of the aorta (CoA), aortic dysplasia and ventricular septal defect (VSD). Prospective ECG-triggering high-pitch DSCT angiography was performed at 80 kV and 60 mAs/rotation (effective radiation dose, 0.24 mSv). (a) Axial multiplanar reformatted image shows VSD. (b) Thin-section oblique sagittal MIP and (c) volume-rendered image (posterior view) show coarctation of the aorta (white arrow) and aortic dysplasia. (d) Two dimensional echocardiography identified CoA, but it missed the aortic dysplasia. RV  =  right ventricle, LV  =  left ventricle, Ar  =  aortic arch, DA  =  descending aorta.

**Figure 4:**
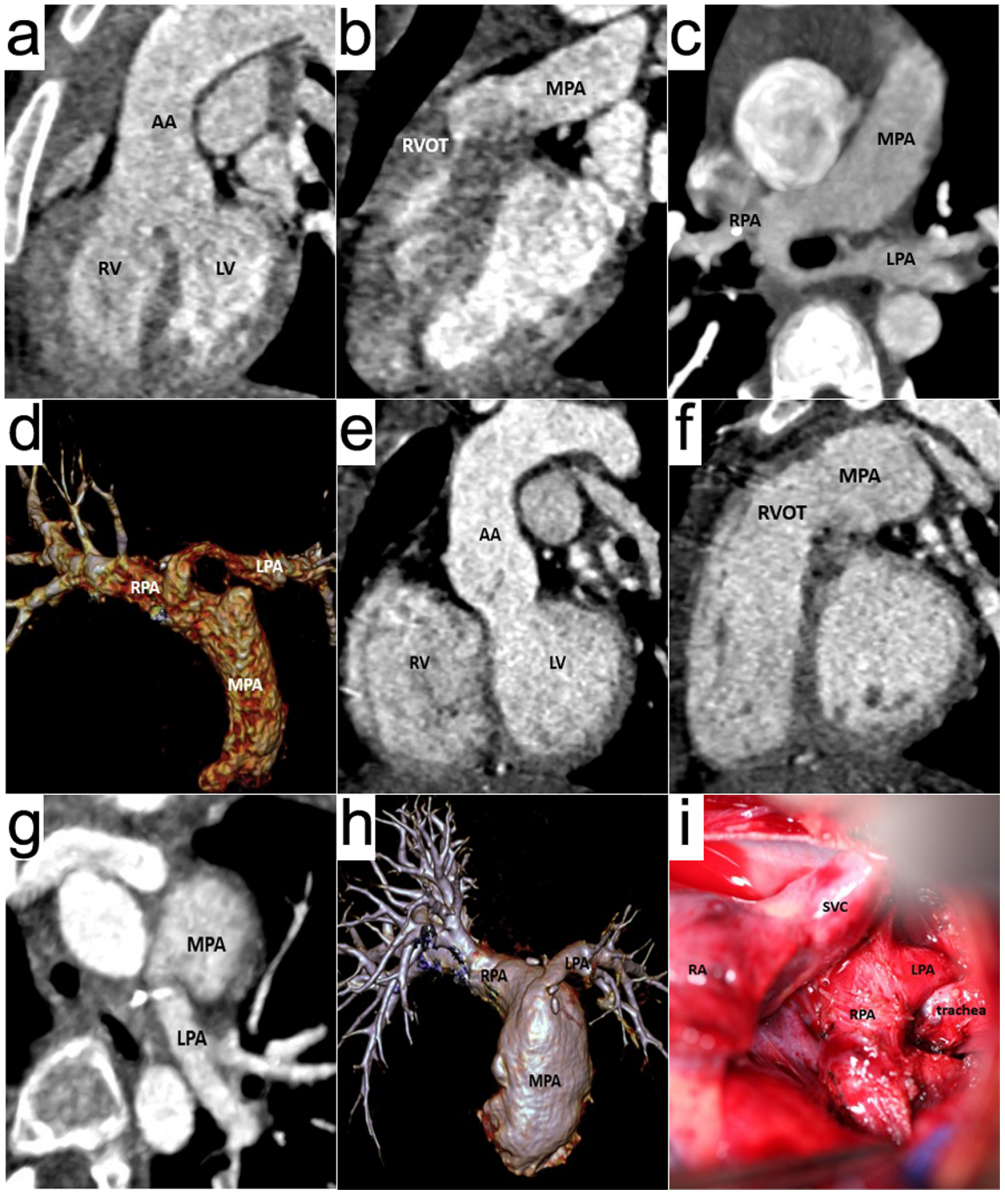
A 5-year girl with Tetralogy of Fallot and pulmonary sling. Prospective ECG-triggering high-pitch DSCT angiography was performed at 80 kV and 100 mAs/rotation (effective radiation dose, 0.29 mSv). (a) Oblique coronal multiplanar reformatted (MPR) image, (b) Oblique sagittal MPR image, (c) axial thin-section MIP and (d) volume-rendered image are before operation, these images show ventricular septal defect (VSD), overriding aorta, right ventricular outflow tract stenosis, pulmonary sling and the stenosis of the initial part of the left pulmonary artery (LPA). (e), (f), (g) and (h) are after operation. (i) is the operation picture. RV  =  right ventricle, LV  =  left ventricle, AA  =  ascending aorta, MPA  =  main pulmonary artery, RPA  =  right pulmonary artery, SVC  =  superior vena cava.

Taking surgical and/or CCA findings as the reference standard, 2 BAVs and 1 single coronary artery were not identified by high-pitch DSCT angiography. High-pitch DSCT angiography misdiagnosed a normal arterial ligament as PDA and a severe pulmonary artery stenosis as pulmonary artery atresia. TTE failed to identify 2 PAPVRs, 1 aortic dysplasia, 2 CoAs, 5 pulmonary artery stenoses, 1 dilated pulmonary artery, 4 pulmonary slings, 1 PDA, 8 MAPCAs, 1 PLSVC and 3 coronary artery anomalies. TTE misdiagnosed 1 case of pulmonary artery atresia as truncus arteriosus, 2 cases of absence of one pulmonary artery as pulmonary sling, 1 CoA as IAA (type A) and 1 MAPCA as PDA. Although 3 TAPVRs were accurately diagnosed by TTE, 1 mixed type was misdiagnosed as cardiac type. Table 1 demonstrates the details on separate cardiovascular abnormalities. Four cases of congenital extracardiac vascular anomalies are shown in Figs. 1–4.

**Table 1:** Findings at prospective ECG-gated high-pitch dual-source CT (DSCT) angiography and transthoracic echocardiography (TTE) referring to surgical and/or CCA results (n = 75).

Cardiovascular deformities	DSCT findings				TTE findings				Surgical/CCA results
	TP	TN	FP	FN	TP	TN	FP	FN	
Transposition of the great arteries	2	73	0	0	2	73	0	0	2
Double outlet right ventricle	2	73	0	0	2	73	0	0	2
Overriding aorta	9	66	0	0	9	66	0	0	9
Truncus arteriosus	3	72	0	0	3	71	1	0	3
Partial anomalous pulmonary venous returns	4	71	0	0	2	71	0	2	4
Total anomalous pulmonary venous returns	3	72	0	0	3	72	0	0	3
Aortopulmonary window	2	73	0	0	2	73	0	0	2
Bicuspid aortic valve	0	73	0	2	2	73	0	0	2
Supravalvular aortic stenosis	2	73	0	0	2	73	0	0	2
Aortic dysplasia	5	70	0	0	4	70	0	1	5
Right aortic arch	6	69	0	0	6	69	0	0	6
Double aortic arch	4	71	0	0	4	71	0	0	4
Coarctations of aorta	23	52	0	0	20	52	0	3	23
Interruption of the aortic arch	4	71	0	0	4	70	1	0	4
Pulmonary artery atresia	5	69	1	0	4	70	0	1	5
Pulmonary artery stenosis	9	65	0	1	5	65	0	5	10
Pulmonary artery dilation	20	55	0	0	19	55	0	1	20
Hemitruncus arteriosus	2	73	0	0	2	73	0	0	2
Absence of a pulmonary artery	2	73	0	0	0	73	0	2	2
Pulmonary sling	7	68	0	0	3	66	2	4	7
Patent ductus arteriosus	22	52	1	0	21	52	1	1	22
Major aortopulmonary collateral artery	12	63	0	0	3	63	0	9	12
Persistent left superior vena cava	8	67	0	0	7	67	0	1	8
Coronary artery anomaly	2	72	0	1	0	72	0	3	3
Total	158	1636	2	4	129	1633	5	33	162

TP, true positive detection; TN, true negative detection; FP, false positive detection; FN, false negative detection.

The diagnostic accuracy of high-pitch DSCT angiography and TTE was 99.67% (158+1636/158+2+4+1636) and 97.89% (129+1633/129+5+33+1633), respectively. The sensitivity of high-pitch DSCT angiography and TTE was 97.53% (158/162) and 79.62% (129/162), respectively. There was significant difference regarding to the diagnostic accuracy and the sensitivity between high-pitch DSCT angiography and TTE (χ^2^ = 23.561 and 28.013, P<0.05).

### Image quality assessment

Diagnostic CT images (images graded 3 or more) were obtained in all patients. The mean image quality score was 4.1±0.7, and distributed as score 3 (n = 15, 20%), score 4 (n = 35, 47%) and score 5 (n = 25, 33%). The agreement on the overall image quality scoring between the two observers was excellent (κ = 0.81).

### Radiation dose estimation

The mean CTDIvol was 0.30±0.07 mGy (range: 0.23 mGy-0.55 mGy). The mean DLP was 5.20±1.60 mGy·cm (range: 3 mGy·cm–9 mGy·cm), resulting in a mean effective radiation dose of 0.29±0.08 mSv (range: 0.12 mSv–0.54 mSv).

## Discussion

Our study demonstrates that prospective ECG-gated high-pitch 128-slice DSCT angiography shares high diagnostic accuracy and sensitivity in the assessment of congenital extracardiac vascular anomalies in children in comparison with TTE. With the image quality of DSCT maintained satisfactory, the radiation dose is lowered to 0.29±0.08 mSv.

### The modalities in assessment of congenital extracardiac vascular anomalies

TTE is adopted usually for the initial assessment of children with CHD. TTE combined with Doppler flow imaging has the advantage in the diagnosis of intracardiac deformities. However, as it is limited by the acoustic window, small field of view (FOV) and lower spatial resolution and is obscured by overlying bone and aerated lung, TTE has relative difficulties in visualizing some extracardiac deformities.

CCA can provide both haemodynamic and anatomical information in a single procedure, with high spatial resolution on the images. The major arguments not to use this modality are the fact it is invasive and runs a 1% procedure-related mortality risk in neonates [19], the long procedure time, possible need for sedation and relatively high doses of ionizing radiation.

As a noninvasive and non-ionizing method, MR which can provide both morphologic and functional information has become an effective modality in the assessment of congenital extracardiac anomalies. However, the long acquisition time may necessitate long periods of sedation for small children which is not desirable. In addition, the spatial resolution is also a limiting factor compared to CT or CCA and can limit clear visualization of the smaller anomalies.

MSCT with high spatial and temporal resolution, various image post-processing techniques, no anesthesia needed due to its fast acquisition, is now becoming one of the most valuable and potential cardiovascular examining tools [20]–[23].

### The technique advantages of 128-slice DSCT

Based on the 64-slice DSCT, the recent 128-slice DSCT has increased the gantry rotation time to 280 ms, resulting in a temporal resolution of 75 ms. Such a fast acquisition allows satisfactory image to be acquired in children with relatively high heart rate and reduces breathing artifacts. The concern with CT imaging in pediatrics is the radiation exposure and here the high-pitch DSCT has advantages over retrospective ECG-gated or sequential techniques previously used, by limiting the exposure to a predefined window in the single cardiac cycle which saves significant dose to the patient.

CT offers a high resolution data set that covers both intracardiac and extracardiac anatomies. The data can also be post processed in a variety of ways (MRP, MIP, VR, etc) making it extremely flexible for visualization and surgical planning. The location, relative size and extent of the lesion, the relationship of the lesion to the adjacent structures are better displayed with MPR, VR and MIP images than with axial images. Axial images are sufficient for assessing intracardiac deformities, and three-dimensional VR images can show great vessels in different directions and angles.

### The diagnostic value of 128-slice DSCT

Our study showed the diagnostic accuracy and sensitivity of high-pitch DSCT angiography in the assessment of extracardiac vascular anomalies was 99.67% and 97.53%, respectively. Compared to TTE, the deformities of aorta, pulmonary vessels, aortopulmonary collateral artery, systemic vein and coronary artery were better demonstrated on DSCT.

DSCT is of greater value compared to TTE in evaluation of congenital aortic diseases [24]–[26]. Except for 2 BAVs, other 44 aortic lesions were all identified by DSCT. Due to the short neck of children, overlying bone and aerated lung and the small FOV examined through suprasternal approach, TTE failed to identify 1 aortic dysplasia and 2 CoAs and misdiagnosed 1 CoA as IAA. Seven cases of CoA accompanied with collateral circulation including prominent internal mammary arteries, descending scapular arteries or intercostal arteries which were identified by DSCT were not diagnosed by TTE.

DSCT allows accurate characterization of pulmonary arterial disorders. It is an important tool helpful in demonstrating the connection of pulmonary artery and the right ventricle, defining anatomic relationship between pulmonary arteries and adjacent structures and calculating McGoon ratio to evaluate the development of pulmonary arteries [27]. DSCT misdiagnosed a severe pulmonary artery stenosis as pulmonary artery atresia, other 44 pulmonary arterial anomalies were all accurately diagnosed. As being easily limited by acoustic window and obscured by aerated lung, TTE is not capable enough to identify pulmonary disorders. Furthermore, the visual zone of left pulmonary artery is smaller than its right counterpart as the former vessel runs backward and is partially covered by the lung, and branches of pulmonary arteries within the lung could not been seen on TTE. In this study, TTE was unable to diagnose 5 pulmonary artery stenoses, 1 dilated pulmonary artery and 4 pulmonary slings. It misdiagnosed 1 case of pulmonary artery atresia accompanied by ventricular septal defect as truncus arteriosus, 2 cases of absence of one pulmonary artery as pulmonary sling.

DSCT is the most valuable modality in diagnosis of anomalous pulmonary venous connections by showing the number, route, location and a full view of the abnormal veins [28]. Seven APVRs in this study were all identified by DSCT. On TTE, the vessels in the proximal part are easily to be detected. However, it is difficult to track the abnormal vein far away the heart. TTE failed to identify 2 PAPVRs and misdiagnosed 1 mixed TAPVR as cardiac type.

In cases of pulmonary atresia, absence or stenosis, the pulmonary vascular bed may be supplied by aortic blood flow including MAPCA or PDA. The number, origin and route of MAPCAs can be shown on DSCT regardless of the size of the vessels [29]. Only large collateral vessels can be identified by TTE, and the ones originating from the descending aorta are difficult to be detected on TTE. Eight cases of MAPCAs were not diagnosed on TTE in our study.

Limited by the acoustic window and small FOV, TTE gives poor performance in demonstrating coronary artery anomalies, especially the middle and distal segment lesions. In 3 patients, TTE missed all of the coronary artery anomalies. DSCT failed to identify 1 single coronary artery, as the acquisition window covered R wave, leading to a big pulsation artifact of the heart.

The diagnosis of LPSVC on TTE relies on the detection of coronary sinus dilation. In our study, one case of LPSVC missed by TTE as the left superior vena cava returned to left atrium and the size of coronary sinus was normal.

DSCT has prominent advantage over TTE in demonstrating trachea and bronchus [30]. In vascular ring and slings, anomalous anatomical relationships between vascular and tracheal structures can be shown on DSCT. Compression narrowing of the airways in 4 patients with double aortic arch and 7 patients with pulmonary sling were identified by DSCT.

We have shown that DSCT can provide low dose, diagnostic image quality data for the majority of extracardiac vascular anomalies. However, there are several anatomical anomalies that are difficult to diagnose with certainty, such as the BAV might go unnoticed because the data set of high-pitch DSCT was obtained in one cardiac cycle and the anomalies of the aortic valves could not be assessed, as happened to 2 patients in our study. In contrast, TTE was able to give accurate diagnosis of the 2 BAVs. Both TTE and DSCT are complimentary, and we found it useful to combine image data from the two compared modalities. Using TTE and DSCT in combination gave 99.4% diagnostic accuracy of the extracardiac vascular anomalies. Only one single coronary artery was not identified with both modalities. With an irregular heart rate in the patient, the starting position of the data acquisition in high-pitch DSCT did not correspond to the preselected starting phase, and the acquisition window covered the R wave, leading to severe artifacts of coronary arteries.

### Limitations

Our study has some limitations. First, high-pitch DSCT could not provide hemodynamic and functional information which may be valuable in the assessment of extracardiac vascular anomalies. Second, a large spectrum of congenital vascular anomalies were enrolled in this study, future studies should be focused on a certain disease. Third, TTE was used as the comparison imaging method and future studies should also look at the diagnostic statistics for other modalities and DSCT.

In conclusion, prospective ECG-gated high-pitch 128-slice DSCT angiography with low radiation dose and high diagnostic accuracy has higher sensitivity compared to TTE in the detection of congenital extracardiac vascular anomalies in infants and children.

## References

[1] Hughes D Jr, Siegel MJ (2010) Computed tomography of adult congenital heart disease. Radiol Clin North Am 48(4):817–835. 20705175 10.1016/j.rcl.2010.04.005

[2] Tsai IC, Chen MC, Jan SL, Wang CC, Fu YC, et al (2008) Neonatal cardiac multidetector row CT: why and how we do it. Pediatr Radiol 38(4):438–451. 18259739 10.1007/s00247-008-0761-9

[3] Paul JF, Rohnean A, Sigal-Cinqualbre A (2010) Multidetector CT for congenital heart patients: what a paediatric radiologist should know. Pediatr Radiol 40(6):869–875. 20432005 10.1007/s00247-010-1614-x

[4] Krishnamurthy R (2010) Neonatal cardiac imaging. Pediatr Radiol 40(4):518–527. 20225116 10.1007/s00247-010-1549-2

[5] Krishnamurthy R (2009) The role of MRI and CT in congenital heart disease. Pediatr Radiol (Suppl 2): S196–S204. 10.1007/s00247-009-1166-0 19308385

[6] Young C, Taylor AM, Owens CM (2011) Paediatric cardiac computed tomography: a review of imaging techniques and radiation dose consideration. Eur Radiol 21(3):518–529. 21188593 10.1007/s00330-010-2036-8

[7] Goo HW (2010) State-of-the-art CT imaging techniques for congenital heart disease. Korean J Radiol 11(1):4–18. 20046490 10.3348/kjr.2010.11.1.4 PMC2799649

[8] Flohr TG, Leng S, Yu L, Aiimendinger T, Bruder H, et al (2009) Dual-source spiral CT with pitch up to 3.2 and 75 ms temporal resolution: image reconstruction and assessment of image quality. Med Phys 36(12):5641–5653. 20095277 10.1118/1.3259739

[9] Lell M, Marwan M, Schepis T, Pflederer T, Anders K, et al (2009) Prospectively ECG-triggered high-pitch spiral acquisition for coronary CT angiography using dual source CT: technique and initial experience. Eur Radiol 19(11):2576–2583. 19760421 10.1007/s00330-009-1558-4

[10] Cheng Z, Wang X, Duan Y, Wu L, Wu D, et al (2010) Low-dose prospective ECG-triggering dual-source CT angiography in infants and children with complex congenital heart disease: first experience. Eur Radiol 20(10):2503–2511. 20532783 10.1007/s00330-010-1822-7

[11] Kim JE, Newman B (2010) Evaluation of a Radiation Dose Reduction Strategy for Pediatric Chest CT. AJR Am J Roentgenol 194(5):1188–1193. 20410401 10.2214/AJR.09.3726

[12] Pache G, Grohmann J, Bulla S, Arnold R, Stiller B, et al (2011) Prospective electrocardiography-triggered CT angiography of the great thoracic vessels in infants and toddlers with congenital heart disease: Feasibility and image quality. Eur J Radiol 80(3):e440–445. 21310567 10.1016/j.ejrad.2011.01.032

[13] Paul JF, Rohnean A, Elfassy E, Sigal-Cinqualbre A (2011) Radiation dose for thoracic and coronary step-and-shoot CT using a 128-slice dual-source machine in infants and small children with congenital heart disease. Pediatr Radiol 41(2):244–249. 20821005 10.1007/s00247-010-1804-6

[14] Achenbach S, Goroll T, Seltmann M, Pflederer T, Anders K, et al (2011) Detection of coronary artery stenoses by low-dose, prospectively ECG-triggered, high-pitch spiral coronary CT angiography. JACC Cardiovasc Imaging 4(4):328–337. 21492807 10.1016/j.jcmg.2011.01.012

[15] Wolf F, Leschka S, Loewe C, Homolka P, Plank C, et al (2010) Coronary artery stent imaging with 128-slice dual-source CT using high-pitch spiral acquisition in a cardiac phantom: comparison with the sequential and low-pitch spiral mode. Eur Radiol 20(9):2084–2091. 20397019 10.1007/s00330-010-1792-9

[16] Goetti R, Baumüller S, Feuchtner G, Stolzmann P, Karlo C, et al (2010) High-pitch dual-source CT angiography of the thoracic and abdominal aorta: is simultaneous coronary artery assessment possible? AJR Am J Roentgenol 194(4):938–944. 20308495 10.2214/AJR.09.3482

[17] Nie P, Wang X, Cheng Z, Ji X, Duan Y, et al (2012) Accuracy, image quality and radiation dose comparison of high-pitch spiral and sequential acquisition on 128-slice dual-source CT angiography in children with congenital heart disease. Eur Radiol 22(10):2057–2066. 22592808 10.1007/s00330-012-2479-1

[18] Ben Saad M, Rohnean A, Sigal-Cinqualbre A, Adler G, Paul JF (2009) Evaluation of image quality and radiation dose of thoracic and coronary dual-source CT in 110 infants with congenital heart disease. Pediatr Radiol 39(7):668–676. 19319514 10.1007/s00247-009-1209-6

[19] Tsai IC, Chen MC, Jan SL, Wang CC, Fu YC, et al (2008) Neonatal cardiac multidetector row CT: why and how we do it. Pediatr Radiol 38(4):438–451. 18259739 10.1007/s00247-008-0761-9

[20] Goo HW, Park IS, Ko JK, Kim YH, Seo DM, et al (2003) CT of congenital heart disease: normal anatomy and typical pathologic conditions. Radiographics 23:S147–165. 14557509 10.1148/rg.23si035501

[21] Maldonado JA, Henry T, Gutiérrez FR (2010) Congenital thoracic vascular anomalies. Radiol Clin North Am 48(1):85–115. 19995631 10.1016/j.rcl.2009.09.004

[22] Chung JH, Gunn ML, Godwin JD, Takasugi J, Kanne JP (2009) Congenital thoracic cardiovascular anomalies presenting in adulthood: a pictorial review. J Cardiovasc Comput Tomogr 3 1 Suppl: S35–46. 19203746 10.1016/j.jcct.2008.11.005

[23] Oguz B, Haliloglu M, Karcaaltincaba M (2007) Paediatric multidetector CT angiography: spectrum of congenital thoracic vascular anomalies. Br J Radiol 80(953):376–383. 16687462 10.1259/bjr/47124005

[24] Kimura-Hayama ET, Meléndez G, Mendizábal AL, Meave-González A, Zambrana GF, et al (2010) Uncommon congenital and acquired aortic diseases: role of multidetector CT angiography. Radiographics 30(1):79–98. 20083587 10.1148/rg.301095061

[25] Yang DH, Goo HW, Seo DM, Yun TJ, Park JJ, et al (2008) Multislice CT angiography of interrupted aortic arch. Pediatr Radiol 38(1):89–100. 17965856 10.1007/s00247-007-0662-3

[26] Nie P, Wang X, Cheng Z, Duan Y, Ji X, et al (2012) The value of low-dose prospective ECG-gated dual-source CT angiography in the diagnosis of coarctation of the aorta in infants and children. Clin Radiol 67(8):738–745. 22336668 10.1016/j.crad.2011.12.007

[27] Chandrashekhar G, Sodhi KS, Saxena AK, Rohit MK, Khandelwal N (2012) Correlation of 64 row MDCT, echocardiography and cardiac catheterization angiography in assessment of pulmonary arterial anatomy in children with cyanotic congenital heart disease. Eur J Radiol 81(12):4211–4217. 22959827 10.1016/j.ejrad.2012.08.010

[28] Vyas HV, Greenberg SB, Krishnamurthy R (2012) MR imaging and CT evaluation of congenital pulmonary vein abnormalities in neonates and infants. Radiographics 32(1):87–98. 22236895 10.1148/rg.321105764

[29] Greil GF, Schoebinger M, Kuettner A, Schaefer JF, Dammann F, et al (2006) Imaging of aortopulmonary collateral arteries with high-resolution multidetector CT. Pediatr Radiol 36(6):502–509. 16555039 10.1007/s00247-006-0143-0

[30] Long YG, Yang YY, Huang IL, Pan JY, Wu MT, et al (2010) Role of multi-slice and three-dimensional computed tomography in delineating extracardiac vascular abnormalities in neonates. Pediatr Neonatol 51(4):227–234. 20713287 10.1016/S1875-9572(10)60043-5

